# Cell migration directionality and speed are independently regulated by RasG and Gβ in *Dictyostelium* cells in electrotaxis

**DOI:** 10.1242/bio.042457

**Published:** 2019-06-20

**Authors:** Taeck J. Jeon, Runchi Gao, Hyeseon Kim, Ara Lee, Pyeonghwa Jeon, Peter N. Devreotes, Min Zhao

**Affiliations:** 1 Department of Biology & BK21-Plus Research Team for Bioactive Control Technology, College of Natural Sciences, Chosun University, Gwangju 61452, Republic of Korea; 2 School of life science, Yunnan Normal University, Kunming, Yunnan 650500, China; 3 Department of Cell Biology, The Johns Hopkins University School of Medicine, Baltimore, MD 21205, USA; 4 Departments of Dermatology and Ophthalmology, Institute for Regenerative Cures, School of Medicine, University of California at Davis, CA 95817, USA

**Keywords:** Directionality, Migration speed, Motility, Electrotaxis, *Dictyostelium*

## Abstract

Motile cells manifest increased migration speed and directionality in gradients of stimuli, including chemoattractants, electrical potential and substratum stiffness. Here, we demonstrate that *Dictyostelium* cells move directionally in response to an electric field (EF) with specific acceleration/deceleration kinetics of directionality and migration speed. Detailed analyses of the migration kinetics suggest that migration speed and directionality are separately regulated by Gβ and RasG, respectively, in EF-directed cell migration. Cells lacking Gβ, which is essential for all chemotactic responses in *Dictyostelium*, showed EF-directed cell migration with the same increase in directionality in an EF as wild-type cells. However, these cells failed to show induction of the migration speed upon EF stimulation as much as wild-type cells. Loss of RasG, a key regulator of chemoattractant-directed cell migration, resulted in almost complete loss of directionality, but similar acceleration/deceleration kinetics of migration speed as wild-type cells. These results indicate that Gβ and RasG are required for the induction of migration speed and directionality, respectively, in response to an EF, suggesting separation of migration speed and directionality even with intact feedback loops between mechanical and signaling networks.

## Abstract (precis)

**Summary:** Cell migration directionality and speed are independently regulated by RasG and Gβ, respectively, in electric field-directed cell migration in *Dictyostelium*, suggesting the points of molecular divergence of the two characteristics.

## INTRODUCTION

Directional cell migration is a highly coordinated process of motility (migration speed), directional sensing and polarity. Motility refers to the ability of cells to move around randomly by extending pseudopods. Recent papers suggest that random extension of pseudopodia is driven by spontaneous actin waves propagating through the cytoskeleton (Allard and Mogilner, 2013; Huang et al., 2013). When exposed to external cues, cells determine the direction of movement by sensing the spatial and temporal information of the external signals, referred to as directional sensing, and persistently move toward the direction of the gradient with forming a morphologically and functionally distinct leading and trailing edges, referred to polarity. Directional sensing and polarity establishment are mediated by a system that detects temporal and spatial stimuli and biases motility toward a certain direction (Artemenko et al., 2014; Shi et al., 2013). There have been numerous studies over the past several decades that investigated these processes and several signaling molecules involved in the directional cell migration have been characterized. However, the interrelationships, the coordinate regulation and the underlying molecular mechanisms of these sophisticated processes remain largely unknown.

Most of our understanding of the basic signaling pathways and molecules involved in directional migration is based on the studies on chemotactic amoeboid cells such as the social amoeba, *Dictyostelium discoideum*, and leukocytes. Directional migration up a gradient of chemoattractants is mediated by a series of signaling molecules, which are differentially activated upon ligand binding to G-protein coupled receptors. The signaling molecules including Ras GTPase, PI3K/PIP3, TORC2/PKB, PLA2, Ca^2^^+^ and cGMP/Myosin II are downstream of the receptor/G proteins and guide the local polymerization of F-actin as well as pseudopod extension at the leading edge of cells (Artemenko et al., 2014; Kortholt and van Haastert, 2008).

Electrotaxis is a directional cell migration in an electric field (EF) and occurs in a variety of types of cells, from bacteria to mammalian cells, including *D.* *discoideum*, skin keratinocytes, corneal epithelial cells and osteoblastic cells (Ferrier et al., 1986; Nishimura et al., 1996; Zhao et al., 1996, 2002). Accumulating studies show that directed migration of cells in an EF is involved in several physiological processes including embryogenesis, neurogenesis and wound healing. It has been demonstrated that naturally occurring endogenous EFs guide directional cell migration during development and wound healing. Disruption of the electrical gradient during development results in skeletal and neural abnormalities (Liu and Song, 2014; Zhao, 2009; Zhao et al., 2006). An applied EF is a directional cue that we can accurately control the magnitude and direction, which can be switched on and off at a precise time point, thus providing a precision experimental tool.

The molecular mechanism of electrotaxis is beginning to be revealed. Generally it is thought that the early stages of signal reception and transduction are not shared, but that a major signaling network such as TORC2 and PI3K pathways, which affect the cytoskeleton, are shared between electrotaxis and chemotaxis. *Dictyostelium* cells have only one Gβ subunit, which is essential for all chemotactic responses in *Dictyostelium* (Artemenko et al., 2014; Zhao et al., 2002). However, *Dictyostelium* cells lacking Gβ display significant directional migration in an EF (Zhao et al., 2002), suggesting that there are some chemotaxis- and electrotaxis-specific pathways. Recently a large-scale screening study identified many genes that mediate electrotaxis in *Dictyostelium* and showed that the TORC2-PKB pathway, including PiaA, GefA, RasC, Rip3, Lst8 and PKBR1, is essential for electrotactic responses (Gao et al., 2015). In addition, large-scale analyses of hundreds of *Dictyostelium* mutant strains showed that the defects in directionality did not always coincide with similar defects in migration speed in some strains. Some mutant strains showing a decrease in directedness displayed increased migration speed, while some hyper-responsive mutants did not show an increase in the migration speed. These phenotypes have also been reported in an RNAi screening study using mammalian cells (Nakajima et al., 2015). Knockdown of some ion-channels had a greater effect on directionality compared to speed, while some affected the speed more than the directedness. These results raise a possibility that directionality and migration speed of cells might be separately regulated during directed cell migration in an EF.

*Dictyostelium discoideum* is a well-developed model organism for cell migration and shows strong electrotaxis (Zhao et al., 2002). In this study, using these genetically amenable cells, we investigated the electrotactic responses of cells to an EF, focusing on migration speed and directionality. Our results reveal the temporal changes in migration speed and directionality, separately, and suggest that Gβ and RasG play important roles in the signaling networks that control migration speed and directionality of cells in an EF, respectively.

## RESULTS

### Large-scale screening for electrotaxis phenotypes

Previously, we developed a high-throughput screening technique and performed large-scale screening to find mutants with electrotaxis phenotypes from a collection of 365 *D.* *discoideum* strains with morphological defects (Gao et al., 2015). The phenotypes of the mutants were separately reanalyzed with respect to two chemotactic indexes, directedness and trajectory speed, to get insights into the relationship between directionality and migration speed in directed cell migration in an EF. All the values of directedness and trajectory speed were converted to relative values with a median. The collection of mutants conformed to a normal-distribution curve in the phenotypes of both directedness and migration speed (Fig. S1). The 2-D plot of the phenotypes, which included both the directedness and the speed of the mutants in EF-directed migration, showed that the values of the directedness and the speed of the mutants were evenly distributed independently of each other, suggesting the absence of any distinct co-relationship between the two phenotypes. In this analysis, the upper/lower or left/right cutoff lines were set at 2.5% of the relative migration speed and directedness values. The mutants were categorized into nine groups; groups showing decreased/-normal/-increased directedness and speed, and mutant strains with defects in directedness and migration speed such that they are located outside the cutoff lines in the plot (Fig. S1B,C). The 2-D analysis of the phenotypes of the collection of mutants demonstrates that the defects in the control of directionality are not necessarily linked with those of migration speed, suggesting the possibility that directionality and migration speed of cells might be separately regulated in directed cell migration in an EF.

### *Dictyostelium* cells exhibit specific acceleration/deceleration kinetics of directedness and trajectory speed in response to EFs

**Fig. 1.**
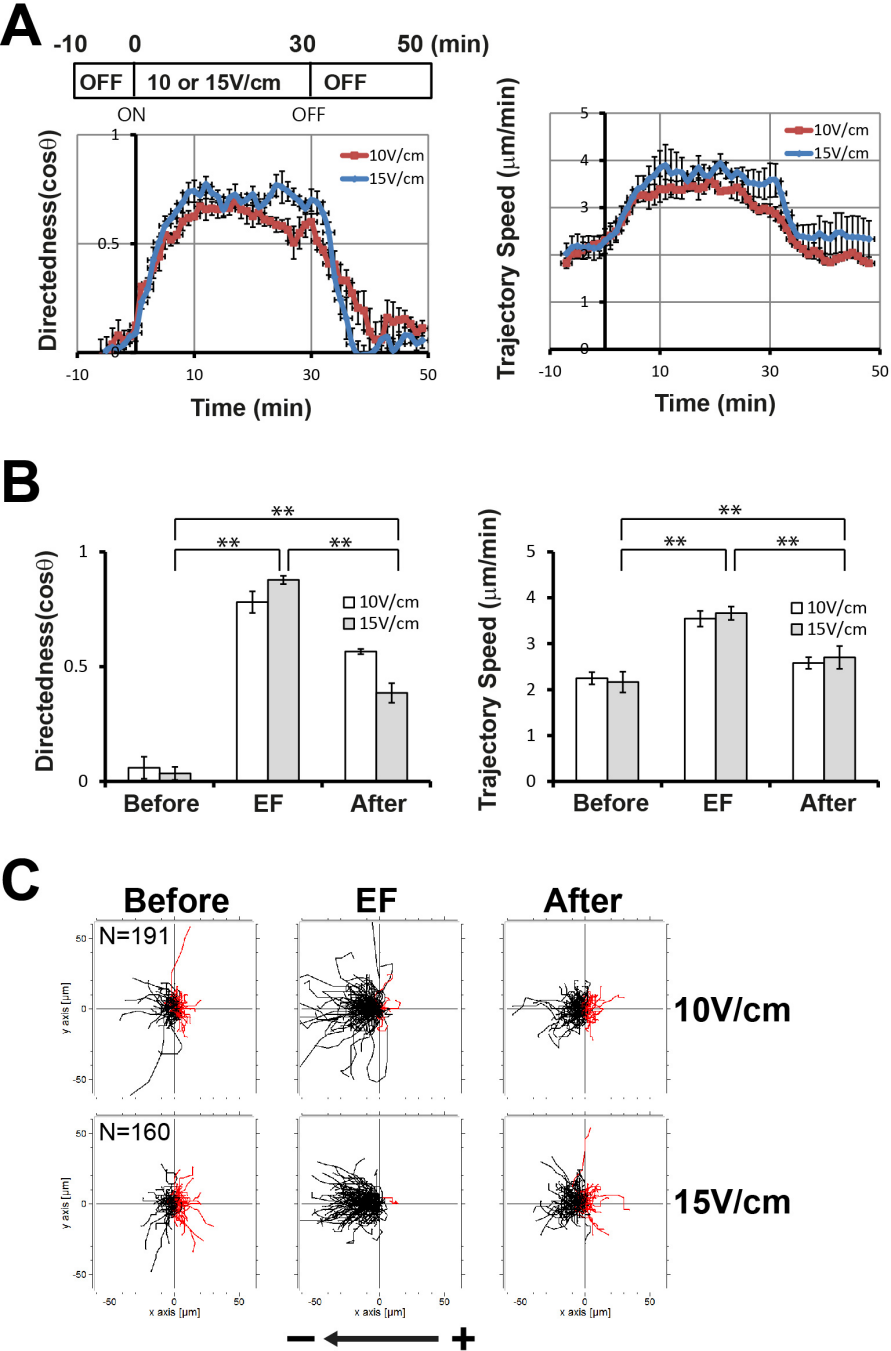
**Electrotactic responses of wild-type Ax3 cells had specific acceleration/deceleration kinetics of directedness and trajectory speed.** (A) Kinetics of directedness and trajectory speed in EF-induced directional migration. Electrotaxing cells were recorded at time-lapse intervals of 1 min for 60 min. No EF was applied for the first 10 min and the last 20 min. Directedness and trajectory speed for every 2 min period were calculated and sequentially plotted. Data are means±s.e.m. from three independent experiments in an EF of 10 V/cm or 15 V/cm. (B) Quantitative analyses of the directional migration of wild-type Ax3 cells in an EF. Directedness and trajectory speed in an EF of 10 V/cm or 15 V/cm were compared with those before applying an EF and after switching off. ‘Before’ indicates the values of directedness and trajectory speed for 10 min right before switching on, ‘EF’ indicates the 10 min after switching on (20 min to 30 min), and ‘After’ indicates the 10 min right after switching off. Statistical analysis was performed using the Student's *t*-test, *P<0.05, **P<0.01. (C) Trajectories of Ax3 cells in an EF of 10 V/cm or 15 V/cm. Plots show migration paths of the cells with the start position of each cell centered at point 0,0. An arrow indicates the direction of an EF. Cells migrate toward the cathode on the left in an EF.

To understand the mechanisms underlying the directed migration of cells in an EF and the relationship between directionality and migration speed in cell migration, we investigated the migration behavior of cells in response to EF stimulation by separately analyzing two indexes of cell movements, directedness of which is for ‘directionality’ and trajectory speed for ‘migration speed’. Directedness and trajectory speed at 2 min intervals were calculated from time-lapse recordings and sequentially plotted (Fig. 1A), along with conventional quantification analyses (Fig. 1B).

Upon exposure to EF, wild-type Ax3 cells showed an immediate response in both directedness and trajectory speed without any time lag. The directedness and trajectory speed gradually increased to the maximum level within 10 min, and remained at the maximum level through the duration of the EF. Within 10 min of turning off the EF, the increased directionality and migration speed gradually decreased back to the basal level (Fig. 1). The kinetics were similar for both directedness and trajectory speed. Other wild-type Ax2 cells also showed similar responses in an EF to those of Ax3 cells (Fig. S2). There was no distinct difference in directional cell migration in an EF between the two different wild-type cells. These results indicate that cells migrate directionally in response to an EF, with specific acceleration/deceleration kinetics of directedness and migration speed.

In agreement with kinetic analyses, the conventional quantification analyses showed that the wild-type Ax3 cells exhibited a strong directional migration toward the cathode in an EF of 10 V/cm or 15 V/cm (Fig. 1B), as reported previously (Gao et al., 2015; Zhao et al., 2002). Before applying EFs, cells moved randomly, indicated by the values of directedness, which were close to 0. The trajectory speed at random movements was approximately 2 μm/min. When the EF was switched on, directedness dramatically increased to 0.8–0.9, and trajectory speed reached to 1.5-fold higher level compared with those before EF exposure. When the EF was turned off after 30 min in the EFs, both indexes of cell migration, directedness and trajectory speed decreased significantly compared with those in the presence of an EF.

### Reversal of the EF polarity results in a complete switch of direction without significant changes in migration speed

**Fig. 2.**
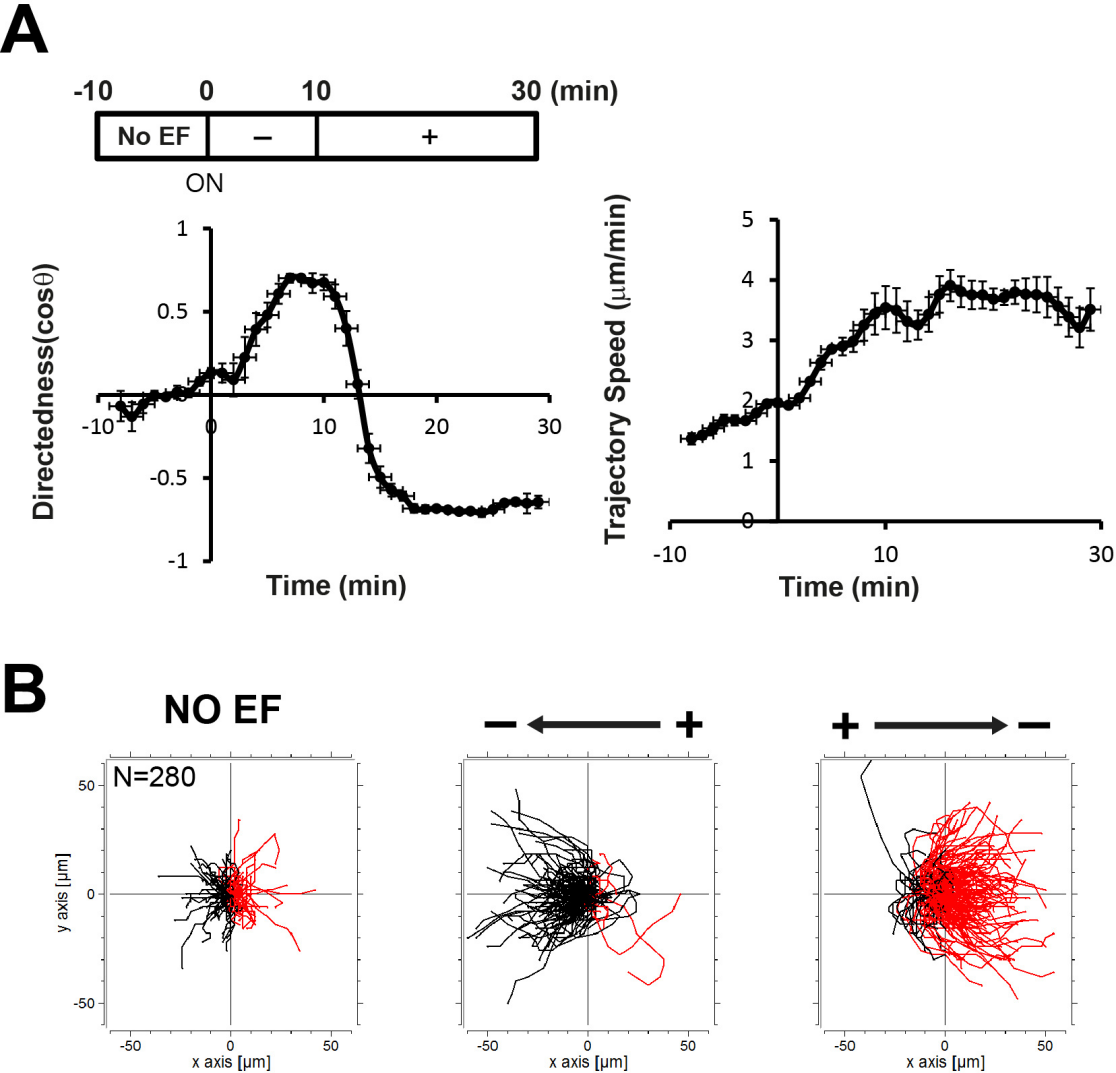
**Effects of reversing EF polarity on cell migration.** (A) Kinetics of directedness and trajectory speed upon reversal of an EF. In 40-min time-lapse recordings, no EF was applied for the first 10 min and the polarity of an EF was reversed at 10 min after switching on. Data are means±s.e.m. from three independent experiments in an EF of 15 V/cm. (B) Trajectories of Ax3 cells for 10 min in the absence of an EF (NO EF), for 10 min in an EF (Cathode), and for 10 min right after reversing the EF polarity (Anode). Plots show migration paths of the cells with the start position of each cell centered at point 0,0. Cathodal and anodal migrations of the cells were marked with black and red, respectively.

To investigate the relationship between directionality and migration speed, we further examined the kinetics of directedness and trajectory speed in EF-directed cell migration by switching the direction of the EF. We first conditioned the cells to maximum directedness and trajectory speed by applying an EF for 10 min, and then quickly reversed the polarity of the EF. Reversal of the EF polarity resulted in complete reversal of the direction of migration within 8–10 min (Fig. 2), a time span similar to that required to attain maximum level upon initial EF stimulation (Fig. 1). By contrast, trajectory speed was maintained at the maximum level with almost no change upon field reversal, if anything there was a transient and small decrease. The trajectories of the cells show a complete directional change upon reversal of the field polarity, but the speed appears similar, indicated by the distance of migration paths of the cells. These results suggest that directionality of cell migration might be regulated independently of migration speed in EF-directed cell migration.

### Gβ null cells are defective in regulating migration speed but not directionality

The binding of the chemoattractant to cell-surface G-protein coupled receptors (GPCRs) initiates the chemoattractant-mediated directional migration. The activated GPCRs promote dissociation of the three subunits of G-protein as Gα-GTP and a Gβγ dimer, both of which mediate downstream signaling pathways for directional cell migration (Artemenko et al., 2014). *Dictyostelium* contains only one Gβ subunit and one Gγ subunit, while 11 Gα subunits have been identified (Artemenko et al., 2014). All chemotactic responses are impaired in cells lacking Gβ subunits in *Dictyostelium* (Parent et al., 1998). In contrast, Gβ null cells display significant directional migration in an EF (Gao et al., 2015). Therefore, the Gβ subunit of G proteins is considered as an important key signaling molecule to differentiate the signaling network of electrotaxis from chemotaxis.

**Fig. 3.**
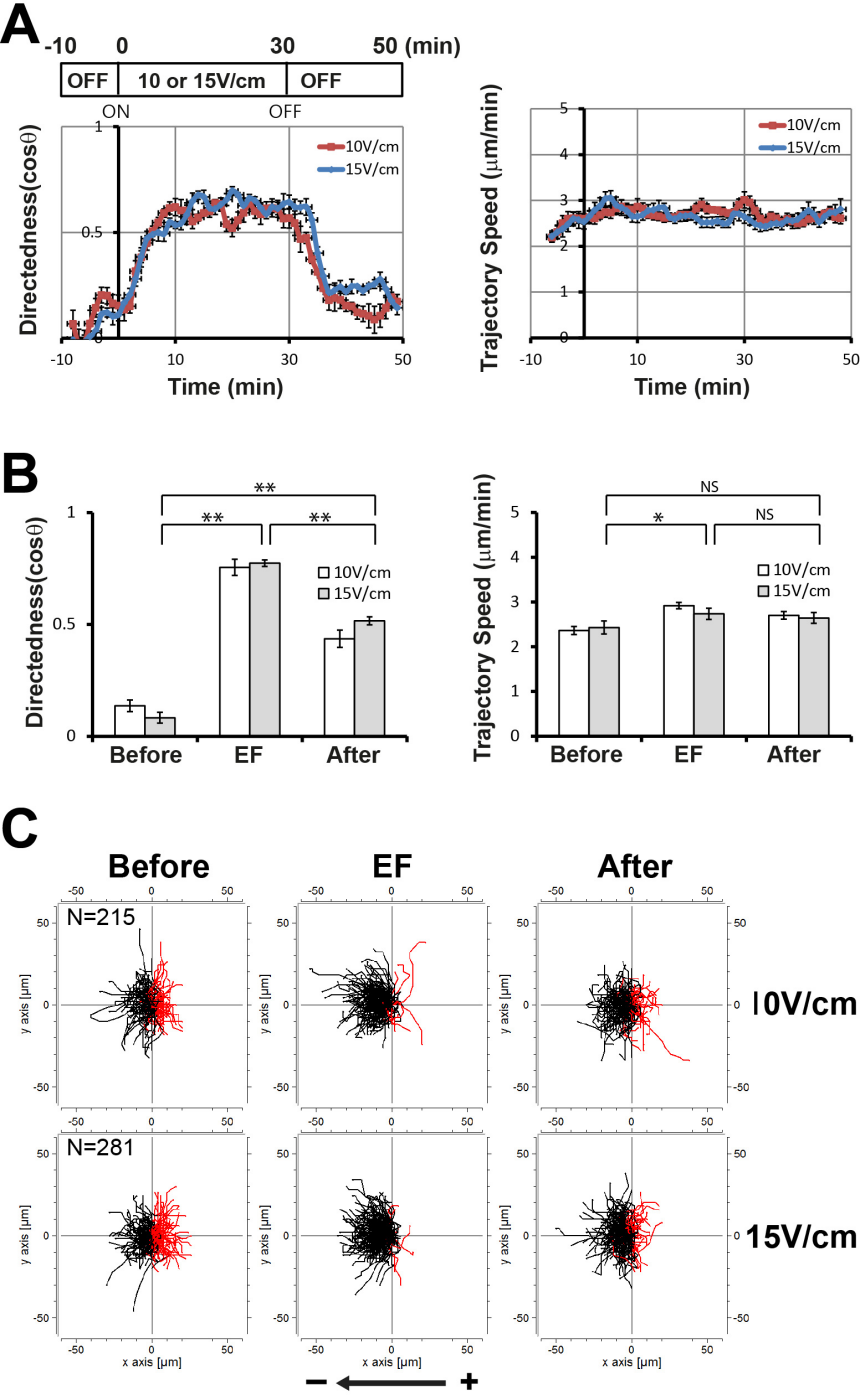
**Electrotactic responses of Gβ null cells.** (A) Kinetics of directedness and trajectory speed of Gβ null cells. Data are means±s.e.m. from three independent experiments in an EF of 10 V/cm or 15 V/cm. (B) Quantitative analyses of the directional migration of Gβ null cells in an EF 10 V/cm or 15 V/cm. *P<0.05, **P<0.01, Student's *t*-test. NS, not significant. (C) Trajectories of Gβ null cells in an EF of 10 V/cm or 15 V/cm.

**Fig. 4.**
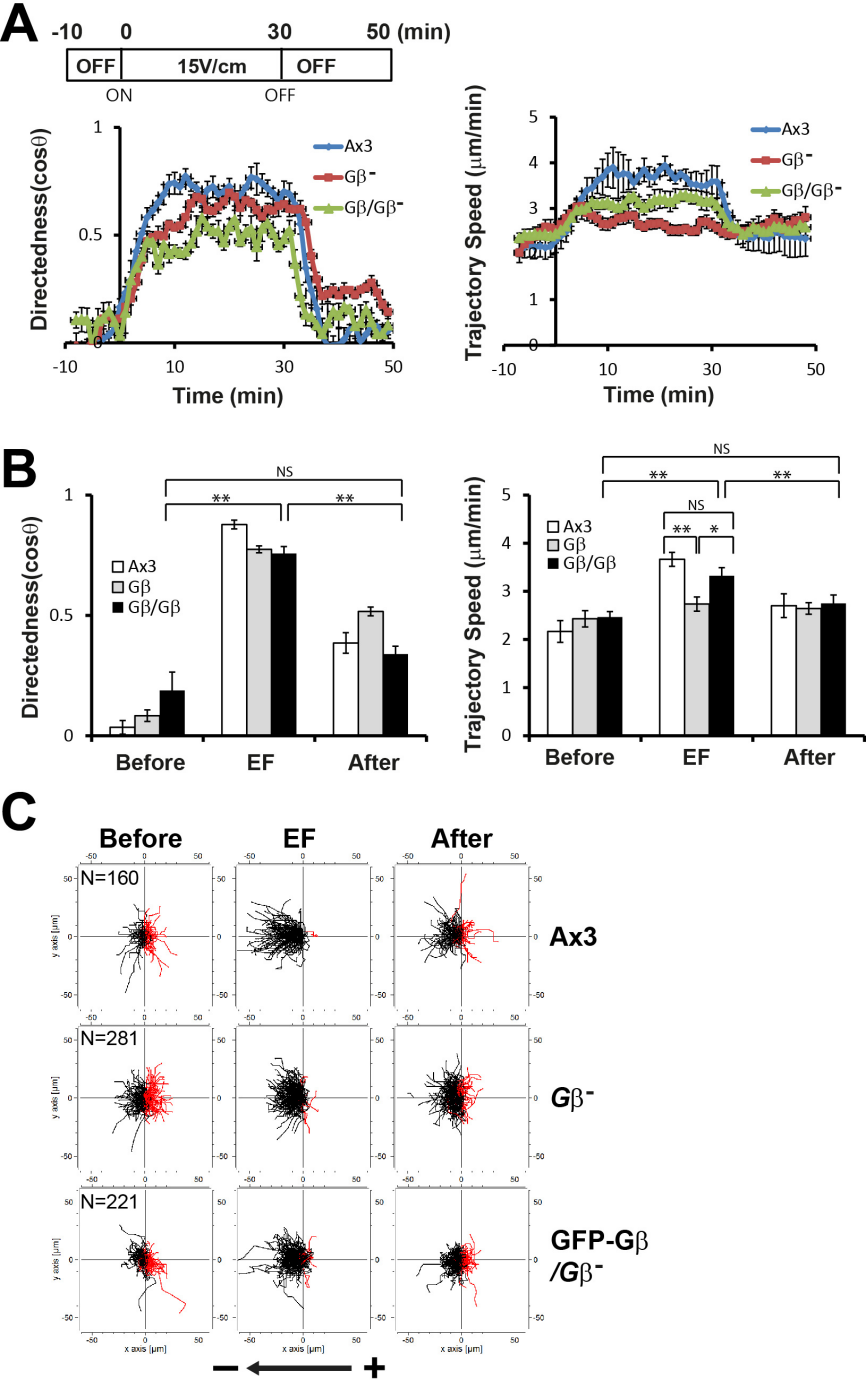
**Comparison of the electrotactic responses of Gβ null cells with wild-type cells and rescue cell strains.** (A) Kinetics of directedness and trajectory speed of Gβ null cells were compared with those of wild-type Ax3 cells and a rescue strain, Gβ null cells expressing GFP-Gβ. Data are means±s.e.m. from three independent experiments in an EF of 15 V/cm. (B) Quantitative analyses of electrotactic responses. Quantitative values of directedness and trajectory speed of Gβ null cells in an EF of 15 V/cm were compared with those of wild-type Ax3 cells and a rescue strain, Gβ null cells expressing GFP-Gβ. *P<0.05, **P<0.01, Student's *t*-test. NS, not significant. (C) Trajectories of Ax3 wild-type cells, Gβ null cells and Gβ null cells expressing GFP-Gβ in an EF of 15 V/cm.

To understand the roles of Gβ in EF-directed cell migration, we examined the kinetics of directedness and trajectory speed upon EF exposure in Gβ null cells (Fig. 3) and compared these with those in wild-type Ax3 cells (Fig. 4). Most strikingly, Gβ null cells did not show the increase in the trajectory speed that both Ax2 and Ax3 (wild type) cells did following exposure to the applied EFs. While wild-type cells displayed a distinct acceleration/deceleration kinetics of trajectory speed upon EF exposure and the significant induction of the trajectory speed (2.2 μm/min before EF exposure) with a maximal speed of approximately 3.7 μm/min within 5–10 min after EF stimulation (15 V/cm) (Fig. 1A), Gβ null cells did not show acceleration of the trajectory speed upon EF stimulation and almost constant migration speed (2.4 μm/min before EF exposure, 2.7 μm/min in an EF of 15 V/cm, 2.6 μm/min after EF turned off) even though there was a slight increase in the initial stimulation of EF (15 V/cm) (Figs 3 and 4, compare with Figs 1A and 2A). The loss of acceleration/deceleration of the trajectory speed in response to an EF in Gβ null cells was rescued by expressing GFP-Gβ (Fig. 4). Gβ null cells expressing GFP-Gβ displayed comparable induction of trajectory speed by EF exposure to the wild-type cells (2.4 μm/min before EF exposure, 3.3 μm/min in an EF of 15 V/cm, 2.7 μm/min after EF turned off). Gβ null cells exhibited directedness kinetics the same as that of wild-type cells: a gradual increase upon EF exposure with a maximum level within 5–10 min and then a decrease to the basal level (Figs 3A and 4A). These results suggest that Gβ is dispensable to EF-induced directionality of cell migration but is essential in mediating EF-induced speed increase. The Gα2 subunit among 11 Gα subunits together with the Gβγ complex plays a critical role in cAMP-directed cell migration during multicellular development in *Dictyostelium*. Gα2 null cells displayed EF-directed cell migration with the similar increase in directedness in an EF as wild-type cells and Gβ null cells (Fig. S4). However, like Gβ null cells these cells failed to show an increase in the migration speed upon EF stimulation as much as wild-type cells.

### *rasG* null cells have a defect in regulating directionality but not migration speed

**Fig. 5.**
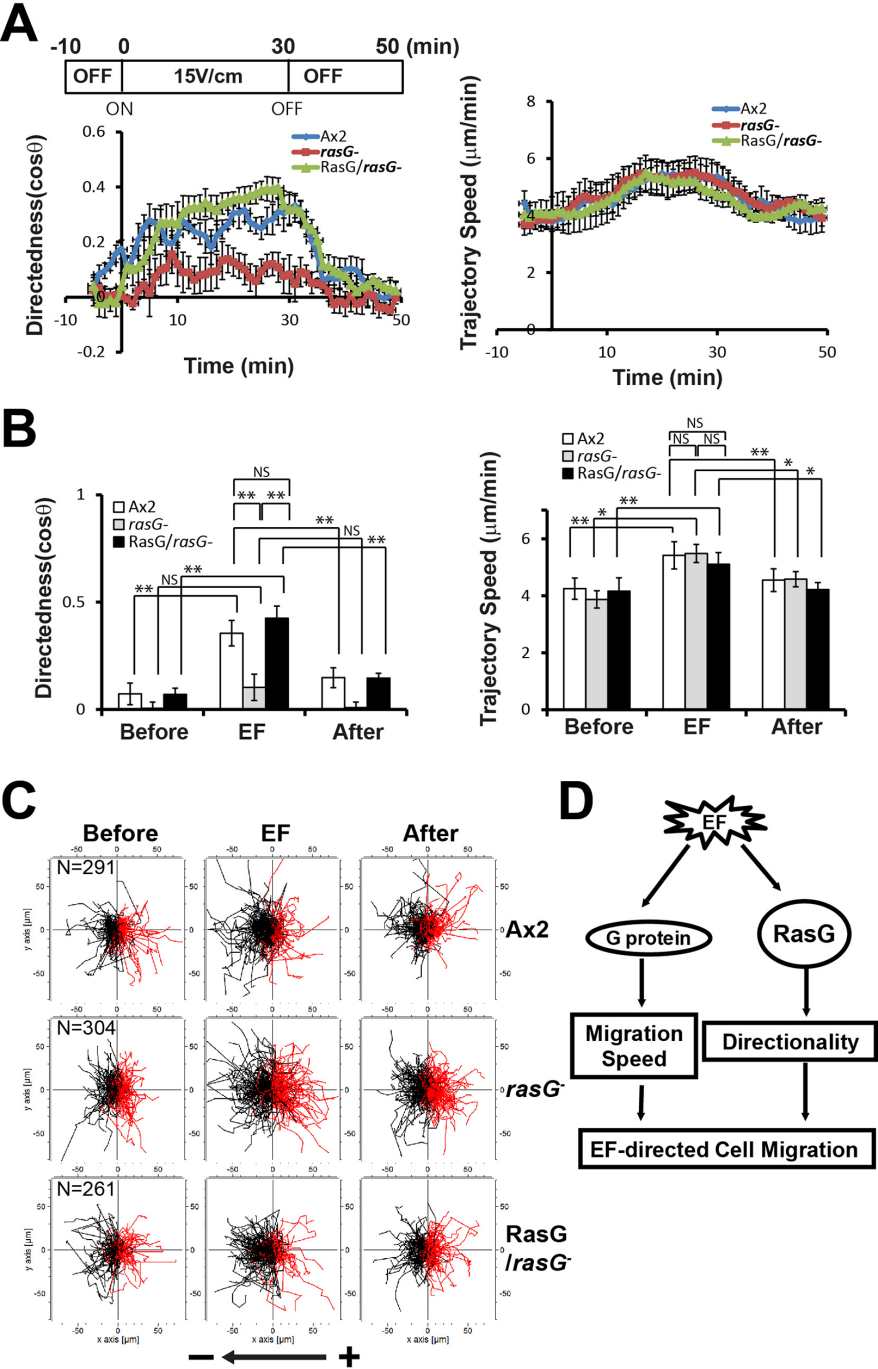
**Electrotactic responses of *rasG* null cells.** (A) Kinetics of directedness and trajectory speed of *rasG* null cells were compared with those of parental wild-type Ax2 cells and *rasG* null cells expressing RasG. Data are means±s.e.m. from three independent experiments in an EF of 15 V/cm. (B) Quantitative analyses of the directional migration of *rasG* null cells in an EF. Quantitative values of directedness and trajectory speed of *rasG* null cells in an EF of 15 V/cm were compared with those of parental wild-type Ax2 cells and *rasG* null cells expressing RasG. *P<0.05, **P<0.01, Student's *t*-test. NS, not significant. (C) Trajectories of *rasG* null cells in an EF of 15 V/cm were compared with those of parental wild-type Ax2 cells and *rasG* null cells expressing RasG. (D) Diagram showing regulation of directionality and migration speed by Gβ and RasG in EF-directed cell migration.

In our large-scale screening study (Gao et al., 2015), a mutant strain that had an insertion site within the *rasG* gene showed a defect in directionality (directedness of 0.31 in an EF). Therefore, we examined migration directionality and speed in cells lacking RasG (Fig. 5). *rasG* null cells exhibited an almost complete loss of directedness. Quantification of the directedness in an EF showed that directedness in *rasG* null cells (0.17±0.05) was significantly lower than in wild-type cells (0.36±0.06). These phenotypes in *rasG* null cells were recovered by expressing Flag-tagged RasG in *rasG* null cells. *rasG* null cells expressing RasG had relatively high values of directedness (0.42±0.06) in an EF, similar to the wild-type cells. The electrotactic response of the trajectory speed upon EF stimulation was similar among these strains, wild-type cells, *rasG* null cells and *rasG* null cells expressing Flag-RasG. *rasG* null cells exhibited kinetics of trajectory speed the same as that of the wild-type cells (Fig. 5). These results indicate that RasG is required for the induction of directionality, but not migration speed, in response to an EF, suggesting the points of molecular divergence of the migration speed and directionality in EF-directed cell migration.

### Inhibition of Ras proteins in the presence of FTS results in a decrease of directionality

**Fig. 6.**
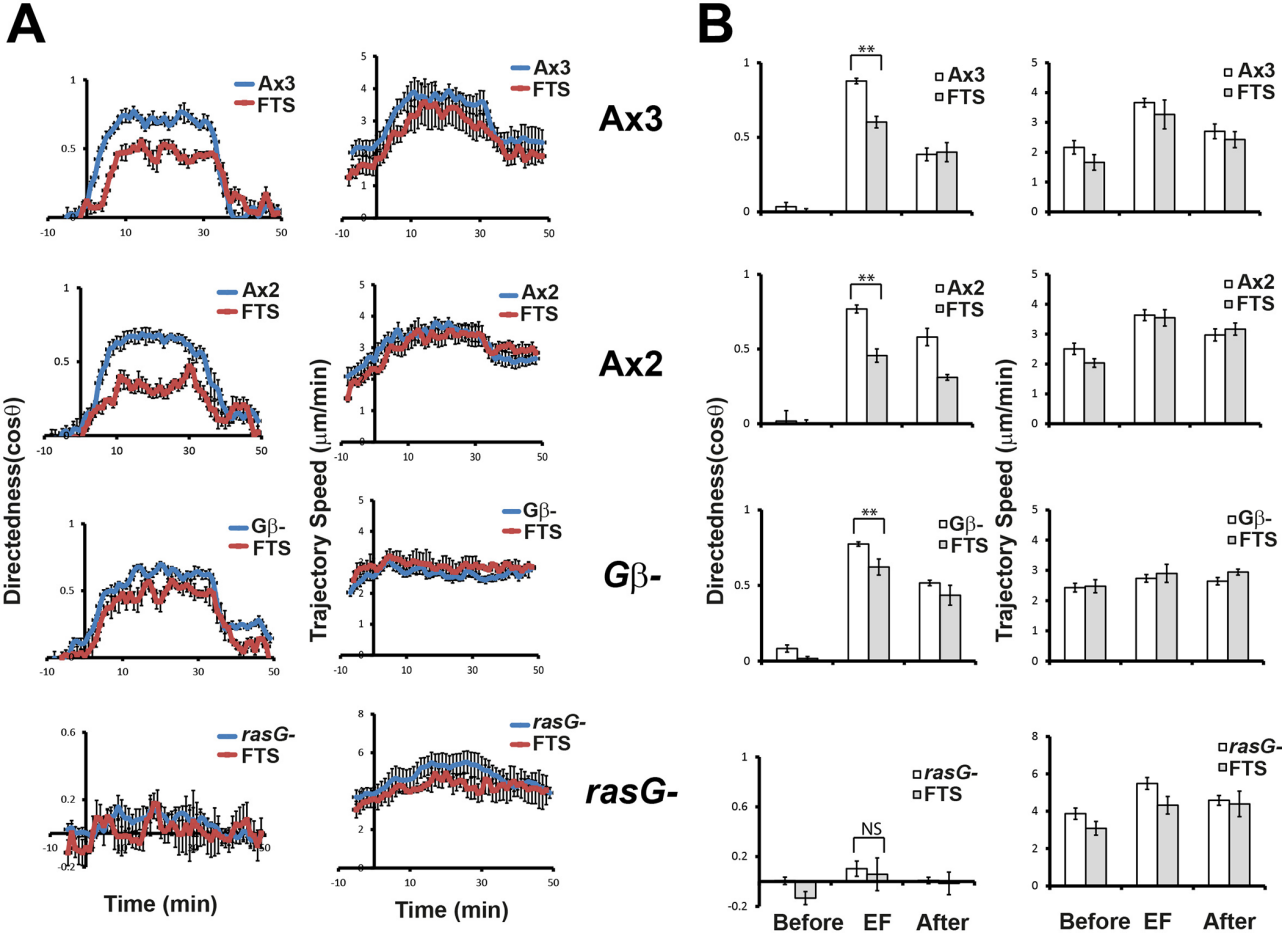
**Electrotactic responses in the presence of FTS.** (A) Kinetics of directedness and trajectory speed in the presence of FTS were compared with those of control cells. Data are means±s.e.m. from three independent experiments in an EF of 15 V/cm. (B) Quantitative analyses of the directional migration in an EF. Directedness and trajectory speed in the presence of FTS were compared with those of control cells. Data are means±s.e.m. from three independent experiments in an EF of 15 V/cm. *P<0.05, **P<0.01, Student's *t*-test. NS, not significant.

To confirm the role of Ras proteins, we examined the electrotactic responses of the cells in the presence of an inhibitor for Ras proteins, farnesylthiosalicylic acid (FTS). FTS, a synthetic farnesylcysteine mimetic, is known to interfere specifically with the functions of the farnesylated Ras proteins by dislodging all farnesylated Ras isoforms from putative Ras anchorage domain in the cell membrane and facilitating its degradation (Haklai et al., 1998). In the presence of FTS, both wild-type Ax3 and Ax2 showed decreased directedness in an EF of 15 V/cm, while there was no significant difference in the trajectory speed compared to the control cells (Fig. 6).

## DISCUSSION

### Kinetics of directionality and migration speed in electrotaxis

We demonstrate that *Dictyostelium* cells migrate directionally towards the cathode with specific acceleration/deceleration kinetics of directionality and migration speed in response to an EF. Our present results show not only that electrotactic responses occur immediately after applying the EF, but also that the two key migration indexes, directionality and migration speed, were gradually increased to the maximal level within 10 min in an EF and remained at the maximum level as long as an EF was being applied. After turning an EF off, directionality and speed – the electrotactic indexes – decreased to the basal level with a slight time lag of approximately 10 min.

Compared with other general cell migration assays, electrotaxis experiments have several advantages in controlling the time for exposure of the cells to the EF and changing the direction of the field polarity. In electrotaxis experiments we can control the strength of the EF accurately and easily, and more importantly, the exact time when the EF starts to be applied or turned off can be controlled, enabling us to assess acceleration/deceleration kinetics of migration indexes in response to EF exposure. Even though many conventional studies on electrotaxis or chemotaxis clearly show a robust induction of directedness and migration speed upon EF exposure or chemoattractant stimulation, the response kinetics of electrotaxis has not been reported. Here we have found that directionality and migration speed of the cells in response to an EF are highly induced with specific acceleration/deceleration kinetics, which allows us to study the molecular mechanisms underlying electrotaxis and directional migration.

### Separate regulation of directionality and migration speed

These results suggest that directionality of the electrotactically moving cells is regulated independently of migration speed. Consistently, recent large-scale mutant screening studies for identifying genes that mediate electrotaxis reported that a decrease or increase of directionality did not always coincide with those of migration speed in some strains (Gao et al., 2015). Our 2-D analysis of the phenotypes of a collection of mutants with the directedness and migration speed demonstrates that the defects on the control of the directedness are not necessarily linked with those in the migration speed, supporting that directionality and migration speed of the cells might be separately regulated in directed cell migration in an EF.

Another group demonstrated that the direction of migration in an EF could be reversed with no effect on migration speed by genetically modulating the genes in cGMP signaling pathway (Sato et al., 2009). Separate regulation of directional sensing and migration speed has been suggested in several chemotaxis studies. The cells of which, where migration speed was inhibited by treatment with an inhibitor of actin polymerization, are still capable of sensing the extracellular chemoattractants and asymmetrically activating signal molecules such as Ras proteins and PIP3 production (Parent et al., 1998; Sasaki et al., 2007; Zhang et al., 2008). Many signaling molecules mediating directionality in chemotaxis have been identified and characterized (Artemenko et al., 2014; Kortholt and van Haastert, 2008; Swaney et al., 2010). Migration speed of the cells is believed to be controlled by dynamic regulation of the cytoskeleton and adhesion complex turnover (Graziano and Weiner, 2014; Huang et al., 2013; Sasaki et al., 2007). However, the molecular mechanisms for coordinate regulation of directionality and migration speed in electrotaxis have not been studied in detail.

### Signaling molecules for controlling directionality and migration speed in electrotaxis

#### Gβ mediates EF-induced increase in cell migration speed

Analysis of the dynamics of the directionality of cells in an EF support the previous conclusion that Gβ is dispensable for EF-induced migration directionality of *Dictyostelium* cells, in sharp contrast to the essential role of Gβ in chemotaxis (Jin et al., 1998; Wu et al., 1995). What is unexpected is that that Gβ null cells did not show an EF-induced increase in cell migration speed. Successful chemotaxis requires both increased migration speed and sustained directionality. The dynamic roles of the G protein subunits in chemotaxis have been studied using new optogenetic tools that enable light-triggered inhibition of G protein subunits in a selected region of a cell. A gradient of activated Gβγ subunit is sufficient to generate phosphatidylinositol (3,4,5)-trisphosphate (PIP3) gradient and lamellipodia formation, and the Gβγ subunits are required for directional sensing in chemotaxis (O'Neill and Gautam, 2014). However, there have been conflicting reports on the roles of G protein βγ subunits. Some reports suggest that G βγ signaling in neutrophil chemotaxis may be primarily involved in controlling cell migration speed (Kamakura et al., 2013; Nishikimi et al., 2009; Stephens et al., 2008). Our data suggest that Gβ is linked to the signaling events that induce the migration speed of the cells in response to an EF.

Migration speed (motility) is mediated by dynamic regulation of the cytoskeleton and adhesion complex turnover. Recently propagating waves of cytoskeletal proteins through the cytoskeleton were suggested to contribute to motile behaviors of the cells (Allard and Mogilner, 2013; Graziano and Weiner, 2014; Huang et al., 2013). Most of F-actin is asymmetrically polymerized at the leading edges of the electrotaxing cells, driving the cells moving forward, through a Rho family of small GTPases which play essential roles in the reorganization of cortical actin cytoskeleton. It has been demonstrated that F-actin accumulation in Gβ null cells is similar to the wild-type cells in response to an EF (Zhao et al., 2002) in the analysis of distribution of coronin-GFP, an F-actin localization marker protein in chemotaxis (Sasaki et al., 2007). Even though the distribution of F-actin polymerization in Gβ null cells upon EF exposure seems normal, further investigation of F-actin polymerization and wave-like propagation of the cytoskeletal proteins in Gβ null cells would be helpful in understanding the roles of G proteins in the regulation of migration speed in EF-directed cell migration.

#### RasG mediates EF-induced cell migration directionality

Contrary to the functions of Gβ in migration speed regulation, RasG appears to be involved in the regulation of directionality but not migration speed. RasG is a key signaling molecule transmitting the signals from G protein/receptor to downstream effectors including PI3K pathways and TORC2 complex for regulating the cytoskeleton in *Dictyostelium* chemotaxis (Kortholt and van Haastert, 2008). *rasG* null cells showed an almost complete loss of directionality and highly increased migration speed compared to wild-type Ax3 and Ax2 cells. Based on this first observation, it was hypothesized that RasG plays some role in the regulation of both directionality and migration speed of the cells in an EF. However, the next confirmation experiments using the parental wild-type Ax2 cells, which were used as background strains for knocking out *rasG*, revealed that removal of RasG resulted in a loss of directionality but no effect on migration speed in directional cell migration in an EF (Fig. S3). Different from other wild-type Ax3 and Ax2 cells, the parental Ax2 strains had highly elevated migration speed compared to other wild-type strains in the absence or presence of an EF. The increased levels and kinetics of migration speed in this parental Ax2 strain were similar to those in *rasG* null cells. However, the parental Ax2 strain showed significantly higher directedness than cells lacking RasG, despite their relatively lower directionality compared to other wild-type strains. Furthermore, this phenotype of *rasG* null cells was rescued by expressing RasG (Fig. S3). These results indicate that RasG is involved in the regulation of directionality but not migration speed in response to an EF.

Cells lacking RasG show decreased chemotaxis index (directionality) and RasG is required for directional cell migration in shallow gradients of chemoattractants (Bolourani et al., 2010; Chattwood et al., 2014). Sasaki et al. reported some conflicting results that *rasG* null cells had slightly increased speed and directionality in chemotaxis and *rasG* null cells expressing dominantly negative RasG stopped motility (Sasaki et al., 2004). Our results suggest that RasG is required for the induction of directionality, but not migration speed, in response to an EF. It has been conceptually suggested that there are two sources to induce directional cell migration; intrinsic cell directionality of migration and external regulation (Petrie et al., 2009). When the cells have a non-directional motogenic signal such as platelet-derived growth factor (PDGF) applied to them, the basic motility machinery is triggered. For directed migration, the motogenic stimulus should be presented to the cells as an external gradient. Even though we do not know the exact mechanism by which RasG regulates directionality of the migrating cells in an EF but not migration speed, one possibility is that EF might provide two types of stimulation as previously suggested by Petrie et al. (2009): one is non-directional and results in a motile response, and the other is directional and drives the cells to move directionally towards the source. RasG might be critical only in sensing directional external, but not non-directional motile stimulation.

Another possibility is that loss of directionality of *rasG* null cells in electrotaxis might result from misregulation of cGMP and cAMP production in response to an EF. Several members of the Ras small GTPase family are expressed in *Dictyostelium*. Five Ras proteins, which share similarities with mammalian H-Ras and K-Ras, have been characterized including RasB, RasC, RasD, RasG and RasS, and their functions partially overlap (Kortholt and van Haastert, 2008). Especially RasC and RasG proteins, which appear to play important roles in directional cell migration. RasC plays a role as an upstream regulator for TORC2 complex in chemotaxis (Charest et al., 2010), and recently it has been shown that cells lacking RasC displayed decreased values of both directionality and migration speed in electrotaxis compared to wild-type cells (Gao et al., 2015). In contrast, RasG appears to be more important in regulating PI3K and cGMP signaling and subsequently is essential for cell polarity and actin polymerization at the leading edge of the cells in *Dictyostelium* chemotaxis (Artemenko et al., 2014; Bolourani et al., 2006; Kortholt and van Haastert, 2008). Disruption of *rasG* in *Dictyostelium* resulted in defects in production of cGMP and cAMP, and activation of Akt/PKB in response to chemoattractant stimulation (Bolourani et al., 2006; Tuxworth et al., 1997). Recently it was demonstrated that the cGMP signaling pathway is involved in controlling the direction of migration in electrotaxing *Dictyostelium* cells (Sato et al., 2009). Studies using *Xenopus* spinal neuron growth cones showed that cAMP/cGMP-dependent modulation of Ca^2+^ channels is important in determining the polarity of the guidance response (Erdogan et al., 2016). Taken together, our results raise a possibility that the control of directionality by RasG in electrotaxis might be mediated through regulating cGMP and cAMP production in response to an EF.

### Conclusion

For directional migration of the cells, two putative signaling networks may be required: one to determine the direction of movement, and the other to regulate the migration speed. We investigated detailed kinetics of directionality and speed of cell migration and found that directionality and speed of migration of the cells in response to an EF are separately regulated by Gβ and RasG, respectively. Gβ is required for an EF-induced increase of cell migration speed, whereas RasG is required for EF-induced directionality.

## MATERIALS AND METHODS

### Strains and cell culture

*Dictyostelium discoideum* cells were obtained from the Dictyostelium Stock Center; wild-type Ax3 cells (DBS0236487), wild-type Ax2 cells (DBS0237914, Jeff Williams lab strain), wild-type Ax2 cells (DBS0235526, Gerry Weeks lab strain), and *rasG* null cells (DBS0236862). A Flag-tagged RasG plasmid was also obtained from the Dictyostelium Stock Center. Gβ null cells and a RFP-tagged LimE plasmid were from Dr Peter Devreotes's lab. All cells were cultured axenically in HL5 medium at 22°C. The knockout strains and transformants were maintained in 10 μg/ml blasticidin or 20 μg/ml G418.

### Cell preparation and electrotaxis

Electrotaxis experiments were performed as described previously (Gao et al., 2015). Growing cells on plates were washed four times and starved for 3 h in development buffer (DB; 5 mM Na_2_HPO_4_, 5 mM KH_2_PO_4_, 2 mM MgSO_4_ and 0.2 mM CaCl_2_), and then subjected to electrotaxis experiments. All procedures were carried out at room temperature (∼22°C). In brief, the prepared cells were seeded on an electrotactic chamber followed by washing off unattached cells with DB buffer. An EF was applied at the indicated field strength through agar salt bridges.

Time-lapse images of cell migration were acquired using an inverted microscope (Axiovert 40, Carl Zeiss) equipped with a charge-coupled device camera (C4742-95; Hamamatsu Corporation) and a motorized XYZ stage (BioPoint 2, Ludl Electronic Products Ltd.) controlled by SimplePCI imaging software.

### Quantitative analysis of electrotaxis

Time-lapse recordings of cell migration with a frame interval of 1 min were analyzed using ImageJ (National Institutes of Health) as previously described (Gao et al., 2015; Zhao et al., 2002). ‘Directedness’ was used to quantify how directionally cells migrated in response to an EF. The directedness of migration was assessed as cosine θ, in which θ was the angle between the direction of the field and a straight line connecting the start and end positions of a cell. A cell moving directly to the cathode or the anode would have a directedness of 1 or −1, respectively. Randomly moving cells would have a value of directedness close to 0. ‘Trajectory speed’ was used to quantify migration speed (motility) of the cells. The trajectory speed is the total distance travelled of a cell divided by time. All motile isolated cells were analyzed from at least three independent experiments. Kinetics of directedness and trajectory speed of the cells were calculated by measuring directedness and trajectory speed of cell migration for every 2 min period in time-lapse recordings and sequentially plotting with time.

### Statistics

Statistical analysis was performed using Student's *t-*tests. All data were expressed as means±s.e.m., and a *P*-value less than 0.05 was considered as statistically significant.

## Supplementary Material

Supplementary information
