# Supplementary material for: Cell migration directionality and speed are independently regulated by RasG and Gβ in Dictyostelium cells in electrotaxis
Source: Biol Open. 2019 Jun 20;8(7):bio042457. doi: 10.1242/bio.042457 (PMC6679393; doi:10.1242/bio.042457)
Supplement: Supplementary information [file biolopen-8-042457-s1.pdf]

A

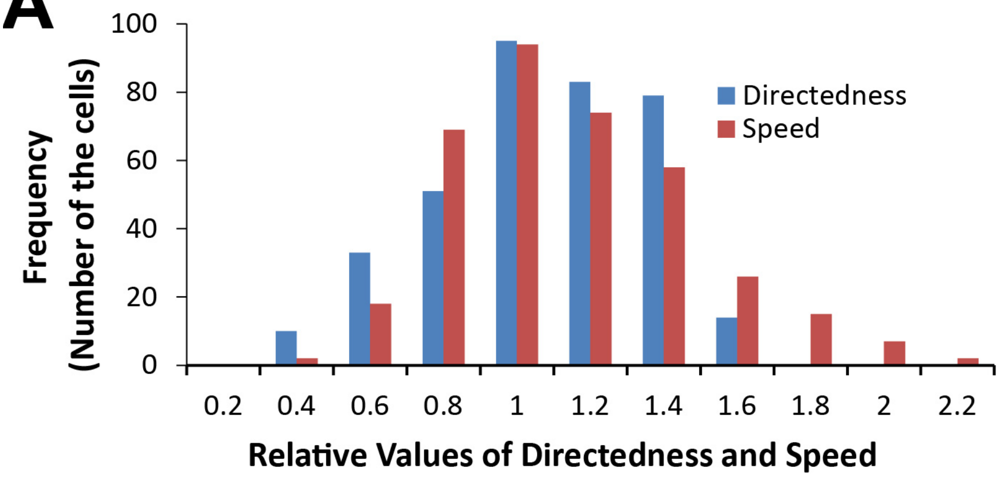

B

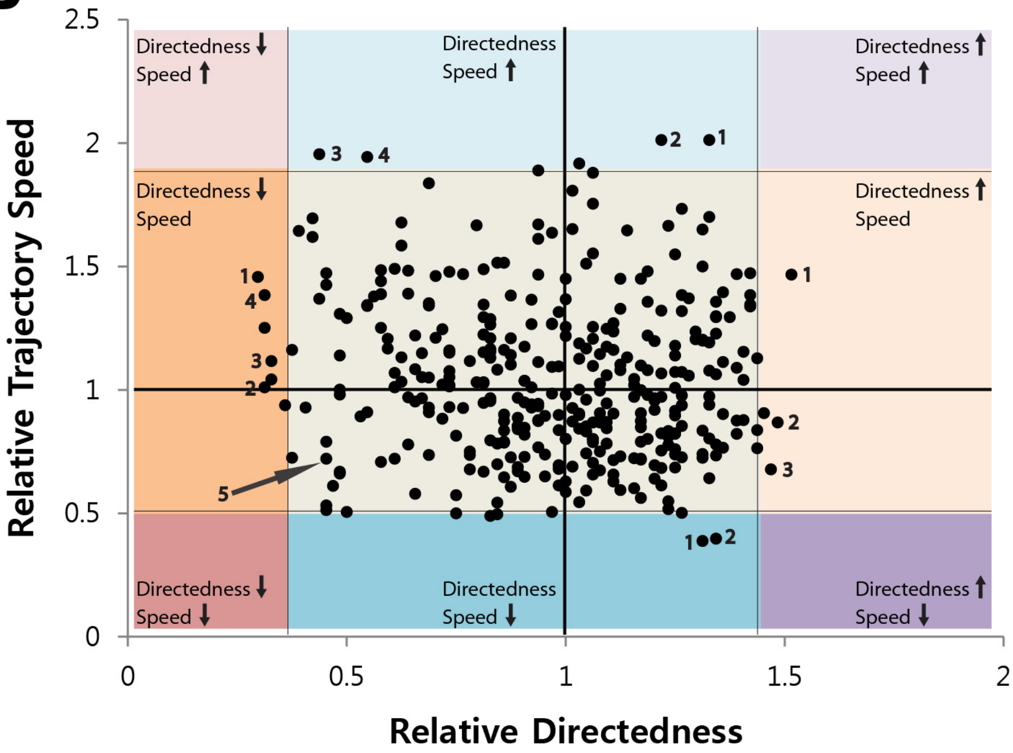

C

	Directedness		Speed	
	Low	High	Low	High
1	SN-494<br>*qtrt1*	SN-563	SN-482	SN-503
2	SN-129<br>*mybl; cmfB*	SN-561	SN-507	SN-405
3	SN-584<br>DDB_G0278163	SN-560		SN-26
4	*piaA*			SN-60
5	*rasG*			

Biology Open • Supplementary information

Fig. S1. Large-scale screen for electrotaxis phenotypes suggests independence between migration directionality and speed.

(A) Histogram of mutants based on the values of directedness or trajectory speed in electrotaxis. The phenotypes of 365 strains from the mutant collection (Gao et al., 2015) were analyzed and plotted. All the values of directedness or trajectory speed were converted to relative values with a median value. The median values of directedness and speed are 0.64 and 5.27μm/min, respectively. (B) Distribution of phenotypes using directedness and trajectory speed in electrotaxis. Relative values of directedness (X axis) of mutants were plotted against trajectory speed (Y axis). The median values of directedness and speed were set to 1 and all other values are relative values. The upper/lower or left/right cut off lines at 2.5% of the migration speed and directedness values were separately drawn, and grouped as 9 groups. Genes located outside of cut off lines were labeled and shown in the following table. (C) Strains showing defects on the directedness and migration speed in electrotaxis. 4 groups are shown, low (left in the plot B)/high (right) in the directedness and low (lower)/high (upper) in the migration speed. The inserted genes in a few REMI mutants were identified and labeled.

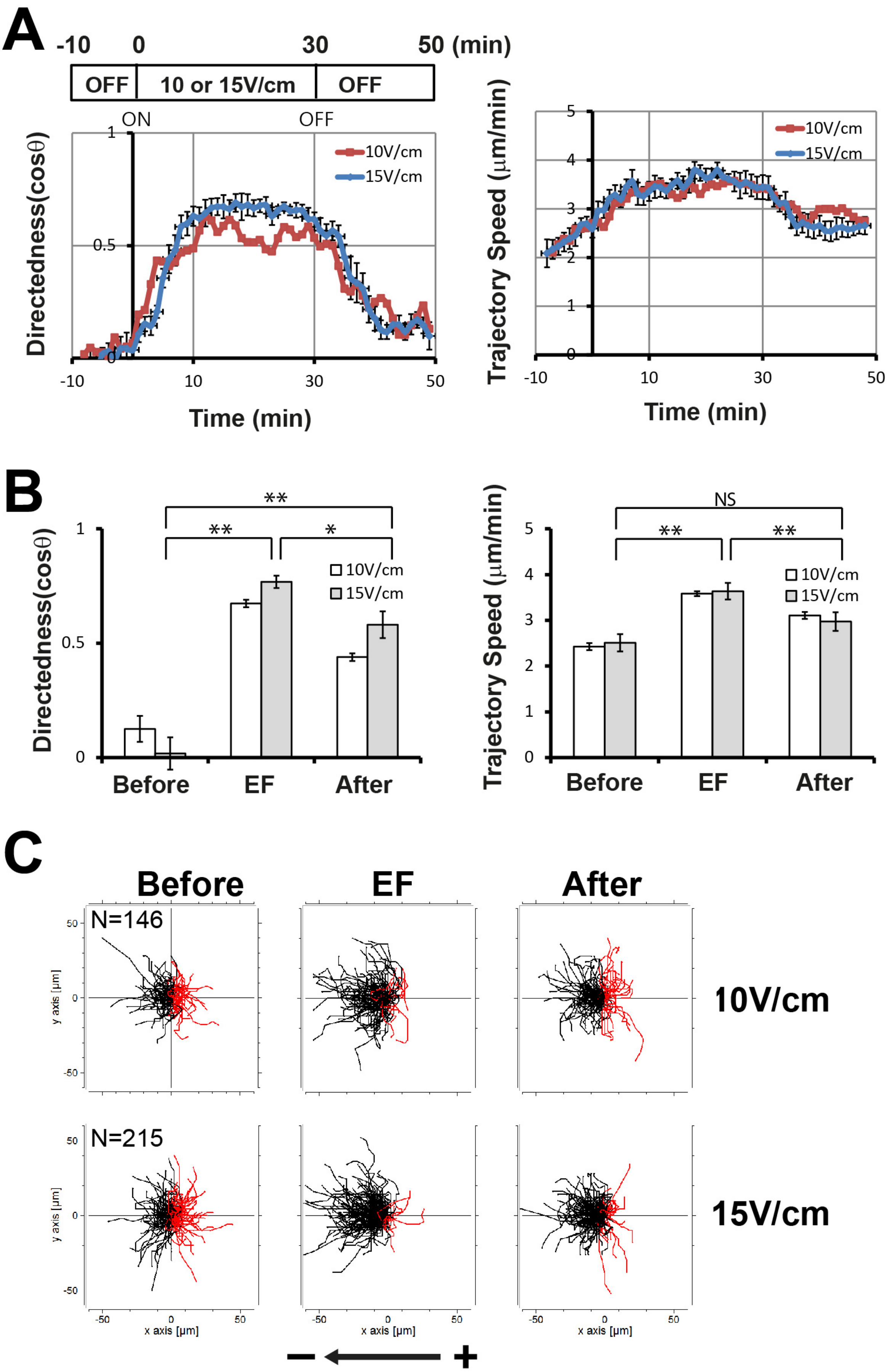

Biology Open • Supplementary information

Fig. S2. Electrotactic responses of wild-type Ax2 cells

(A) Kinetics of directedness and trajectory speed of wild-type Ax2 cells in EF-induced directional migration. Electric field was applied and the data were analyzed as described in figure 1. Data are means ± SEM from three independent experiments in an electric field of 10V/cm or 15V/cm. (B) Quantitative analyses of the directional migration of wild-type Ax2 cells in an electric field. (C) Trajectories of Ax2 cells in an electric field of 10V/cm or 15V/cm.

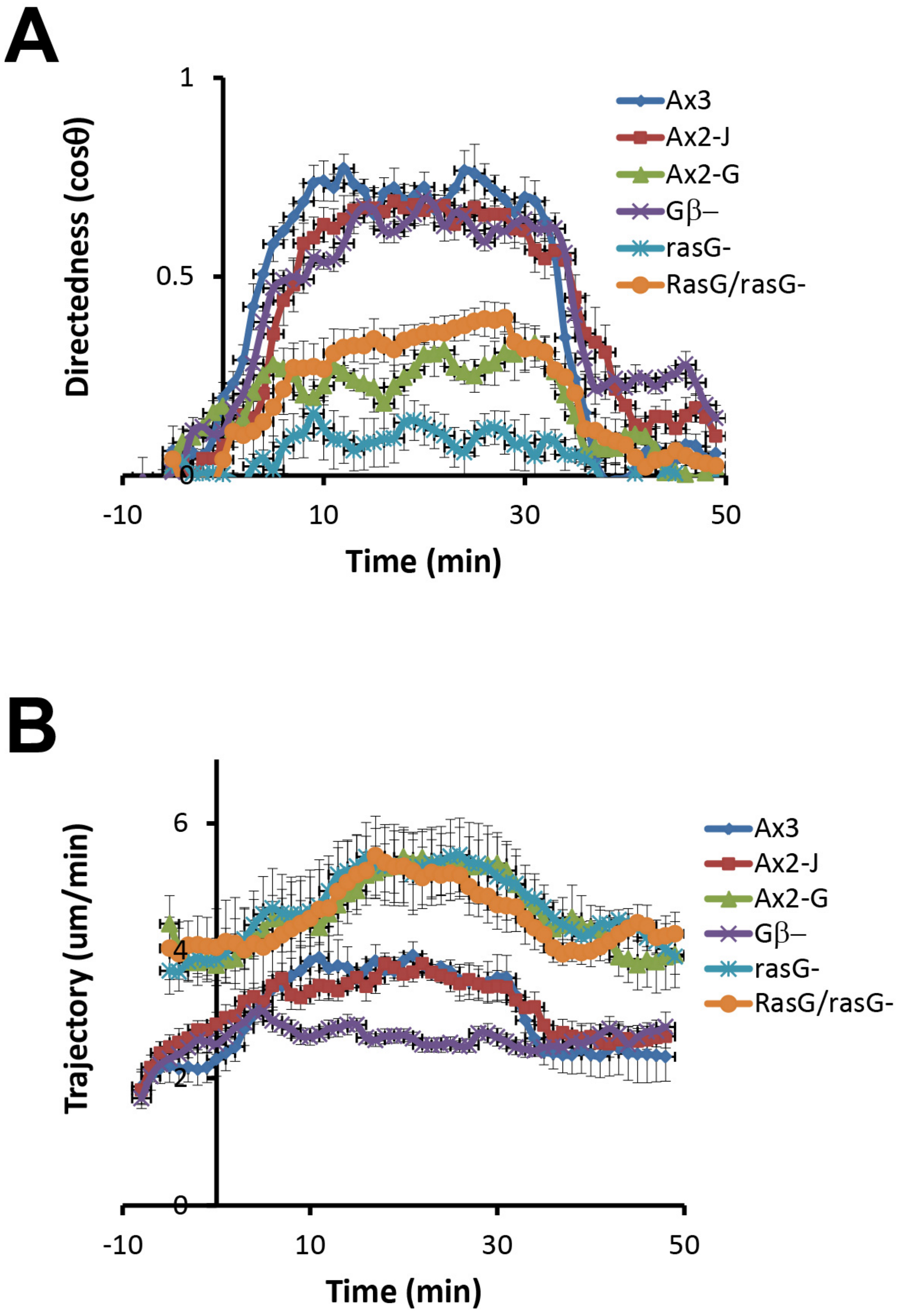
A
Directedness (cosθ)
1
0.5
0
-10
10
30
50
Time (min)
Ax3
Ax2-J
Ax2-G
Gβ–
rasG-
RasG/rasG-
B
Trajectory (um/min)
6
4
2
0
-10
10
30
50
Time (min)
Ax3
Ax2-J
Ax2-G
Gβ–
rasG-
RasG/rasG-

Fig. S3. Kinetics of EF-directed cell migration in different cell strains

(A) Kinetics of directedness in different strains. The parental background strains of Gβ and *rasG* null cells are Ax3 and Ax2-G cells, respectively. Another Ax2-J strains and *rasG* null cells expressing RasG were analyzed as well. Data are means ± SEM from three independent experiments in an electric field of 15V/cm. (B) Kinetics of trajectory speed in different strains. Data are means ± SEM from three independent experiments in an electric field of 15V/cm.

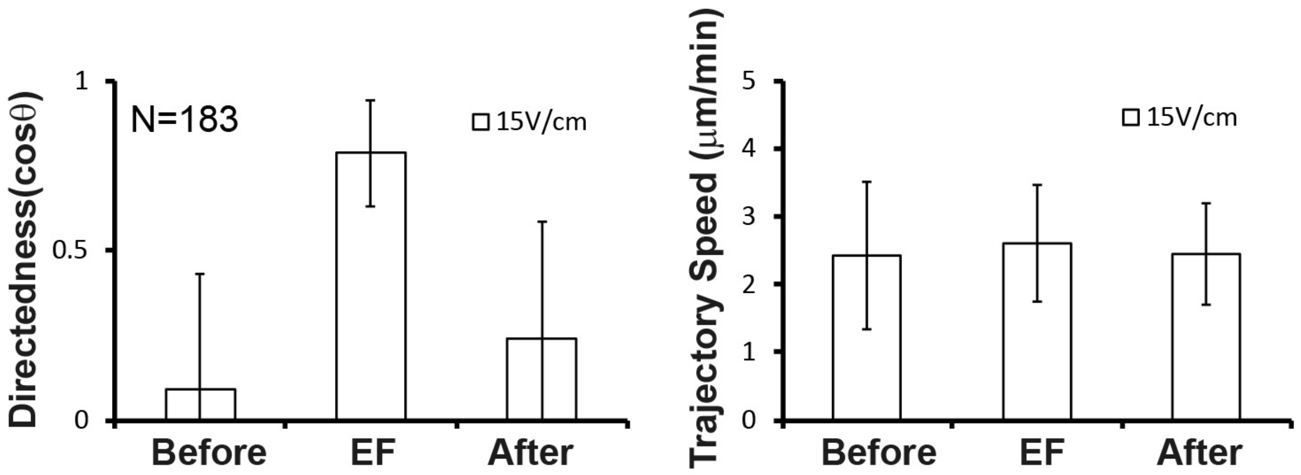

Fig. S4. Electrotactic responses of Gα2 null cells

Quantitative analyses of the directional migration of Gα2 null cells in an electric field. Directedness and trajectory speed in an electric field of 15V/cm were compared with those before applying an electric field and after switching off. 'Before' indicates the values of directedness and trajectory speed for 10 min right before switching on, 'EF' for 10 min after switching on (20 min to 30 min), and 'After' for 10 min right after switching off. The values are the means ± SD of three independent experiments.
